# What to know before forecasting the flu

**DOI:** 10.1371/journal.pcbi.1005964

**Published:** 2018-10-12

**Authors:** Prithwish Chakraborty, Bryan Lewis, Stephen Eubank, John S. Brownstein, Madhav Marathe, Naren Ramakrishnan

**Affiliations:** 1 Discovery Analytics Center, Virginia Tech, Blacksburg, Virginia, United States of America; 2 Department of Computer Science, Virginia Tech, Blacksburg, Virginia, United States of America; 3 Biocomplexity Institute, University of Virginia, Charlottesville, Virginia, United States of America; 4 Network Dynamics and Simulation Science Laboratory, Biocomplexity Institute, Virginia Tech, Blacksburg, Virginia, United States of America; 5 Children's Hospital Informatics Program, Boston Children’s Hospital, Massachusetts, United States of America; 6 Department of Pediatrics, Harvard Medical School, Massachusetts, United States of America; 7 Department of Computer Science, University of Virginia, Charlottesville, Virginia, United States of America; Ecole Polytechnique Federale de Lausanne, SWITZERLAND

## Summary

Accurate and timely influenza (flu) forecasting has gained significant traction in recent times. If done well, such forecasting can aid in deploying effective public health measures. Unlike other statistical or machine learning problems, however, flu forecasting brings unique challenges and considerations stemming from the nature of the surveillance apparatus and the end utility of forecasts. This article presents a set of considerations for flu forecasters to take into account prior to applying forecasting algorithms.

## Introduction

**Fig 1:**
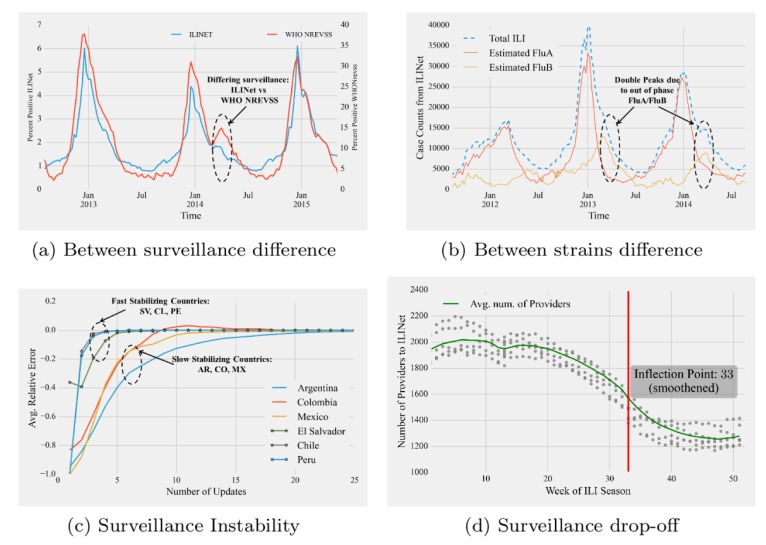
Surveillance characteristics. Data range 2012–2015. (a) Lag between reported ILI percentage curves as reported by two surveillance systems (ILINet versus WHO NREVSS) in the US, (b) Lag between ILI substrain reports at national level (Flu A vs Flu B) leading to double peaked overall ILI curve in the US, (c) Surveillance reports are revised many weeks after first report. While countries like Chile stabilizes quickly (within 5 weeks), other countries like Argentina stabilizes after many weeks (≥10). (d) Surveillance drop-off towards the end of the season—scatter plot of number of providers reporting to CDC ILINet as a function of ILI season week. Green Line shows the smoothened average while the red vertical line shows the smoothened inflection point of surveillance coverage. (Smoothing interval = 4) CDC, Centers for Disease Control and Prevention; ILI, Influenza-like Illnesses; NREVSS, National Respiratory and Enteric Virus Surveillance System.

**Fig 2:**
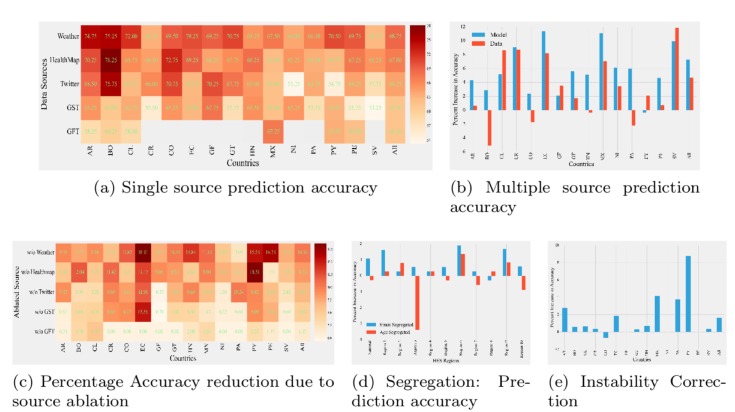
Forecasting characteristics. Data range 2012–2015. (a) *Single Source*: Forecast accuracy for each individual source (Weather, HealthMap, Twitter, Google Flu Trends, and Google Search Trends). No particular source is the best for all countries. (b) *Multiple Sources*: Percent increase in forecast accuracies while combining multiple sources at model level and at data level over best single source forecasts [1]. Model level gives better overall performance. (c) *Ablation test*: Percent reduction in forecast accuracies while removing one source at a time from the fused model. Removing a source can lead to better performance for some countries [1]. (d) *Segregation Test*: Percent increase in forecast accuracies for US ILI data considering forecasts made individually for ILI cases by age and by subtype over forecasts on unsegregated data. Segregated methods show better accuracy. (e) *Instability Correction*: Percent increase in forecast accuracies for different countries after correction over uncorrected forecasts. Significant improvement can be seen for countries like Argentina and Paraguay [1]. ILI, Influenza-like Illnesses.

During the start of every new flu season, we hear the usual cautionary notes about vaccinations, the preparedness of our health systems, and the specific strains that are relevant for the upcoming season. Once considered heterdox, forecasting the characteristics of the annual flu season is now a mainstream activity, thanks to scientific competitions organized by agencies like the Centers for Disease Control and Prevention (CDC; https://www.cdc.gov/flu/news/flu-forecast-website-launched.htm) and Intelligence Advanced Research Projects Activity (IARPA; https://www.iarpa.gov/index.php/research-programs/osi). While the CDC competition aimed to forecast flu seasonal characteristics in the United States, the IARPA Open Source Indicators (OSI) forecasting tournament was focused on disease forecasting (flu and rare diseases) in countries of Latin America. Our team was declared the winner in the IARPA OSI competition and as the winner in one category of the CDC competition (namely, predicting seasonal peak characteristics). While many statistical and machine learning algorithms are now popularly used in this domain (e.g., see [1–3]), flu forecasting brings some unique considerations that necessitate novel data preprocessing and modeling strategies. Our goal here is to distill some of our lessons learned into considerations to take into account, prior to applying a forecasting algorithm. Our focus is thus not on the forecasting algorithm itself but data preprocessing and modeling considerations that all forecasting algorithms must grapple with. These considerations fall in the categories of “Surveillance Characteristics” (see Fig 1) and “Forecasting Methodology” (see Fig 2). More details about the data sets, associated code snippets, figures, and abbreviations used in this article are archived at the publicly accessible webpage http://prithwi.github.io/how_not2_flu.

We discuss these considerations and present our recommendations on pitfalls to avoid below.

## Surveillance characteristics

Flu surveillance networks exhibit several unique traits which can further be modulated by regions/time period of interest. An effective flu forecasting system needs to pay careful attention to such characteristics. We discuss some of the more important, but often overlooked, aspects of the surveillance characteristics in the following sections.

### Surveillance networks do not measure the same quantity

Influenza-like lllnesses (ILI), tracked by many agencies such as the CDC, the Pan American Health Organization (PAHO), and WHO (World Health Organization) [4–6], is a category designed to capture severe respiratory diseases like flu but also includes many other less severe respiratory illnesses due to their similar presentation. Surveillance methods often vary between agencies. Even for a single agency, there may be different networks (such as outpatient based and lab sample based) tracking ILI/flu. While outpatient reporting networks such as the US Outpatient Influenza-like Illness Surveillance Network (ILINet) aim to measure exact case counts for the regions under consideration, lab surveillance networks such as the WHO National Respiratory and Enteric Virus Surveillance System (NREVSS, used by PAHO) seek to confirm and identify the specific strain. In the absence of a clinic-based surveillance system, lab-based systems can provide estimates for the population based on percent positives in the samples; however, making an estimate of actual influenza flu cases from these systems is challenging [4]. Furthermore, surveillance reports are often nonrepresentative of actual ILI incidence (see "epidemic data pyramid" in additional resources) and can often suffer from variations such as holiday periods when behavior of people visiting hospitals changes from other weeks (see "Christmas effect" in additional resources). Additionally, the representativeness of a positive lab sample in a densely populated country with wide lab network coverage may differ from one in a more resource constrained lab surveillance system. For example, in the US, the CDC reports percentage positive ILI for ILINet (an outpatient reporting network) and for WHO NREVSS (based on lab samples). While both networks are designed for different purposes and provide valuable information for forecasting, they exhibit significant lags between reported ILI seasons (see Fig 1A), and it is essential for forecasters to distinguish between surveillance networks and their intricacies. This difference is especially important when conducting cross-correlation studies between different countries (e.g., geographically nearby tropical countries may have similar underlying ILI activity) or even between two different networks for the same country.

### ILI is plural

Many surveillance systems for influenza report on ILI [4], which is based on symptoms of fever (temperature of 100°F or greater) and cough and/or sore throat. This general classification is easier to gather than more expensive and labor-intensive virological tests; however, this definition means that multiple influenza strains and respiratory infections are aggregated together. Such aggregation poses difficulties in modeling because different strains of the flu exhibit different seasonal characteristics (see Fig 1B in which Flu A shows significantly time-shifted seasons than Flu B). Consequently, a monolithic forecasting model that doesn't distinguish between such subcategories is unlikely to capture the behavior of the overall epicurve. Our recommendation is to not treat ILI as an atomic illness and to build models that are cognizant of substrains if data at required resolution(s) are available. Although [7] has reported successful results via monolithic models, our experiments using models from [1] suggest that such breakdown by strains can produce better quality predictions by accounting for temporal shifts between them (see Fig 2D).

### Modeling geographic diversity

Similarly, building models at the national level without considering differences in geography is likely to lead to erroneous results, as ILI characteristics are again shifted and scaled differently across regions. In a geographically diverse country such as the US, ILI seasonal curves are consistently out of phase between the different regions as defined by the U.S. Department of Health and Human Services (HHS) as well as the national curve. Furthermore, a single region (e.g., HHS region 10, which subsumes states such as Alaska and Washington) can be composed of noncontiguous land masses, which may make any assumption about uniformity in ILI profiles inaccurate.

### Surveillance data is not stable

From our experiences/experiments we have found that surveillance data are often delayed and can be candidates for revision/updates for several weeks after initial publication. The lag between initial publication and final revision can be as small as 2 weeks (e.g., for CDC ILINet data) or can wildly fluctuate (e.g., PAHO reports for some Latin American countries such as Argentina, Colombia, and Mexico can take more than 10 weeks to settle. On the other hand, PAHO reports stabilize within 5 weeks for countries such as Chile, Costa Rica, and Peru [see Fig 1C]). The reason for such discrepancies can possibly be due to many complex factors such as maturity of the surveillance apparatus and the level of coordination underlying public health reporting. Most works on forecasting do not account for such revisions in reports (data instability). Some researchers [2, 3] have used empirical methods to factor possible variations in data; however, such empirical methods don't provide guidance for modeling the broader geographical context/surveillance apparatus. In essence, we are forecasting a moving target and recent research [8] suggests that modeling such uncertainty directly in a forecasting model can significantly improve performance. In particular, a stabilization or autocorrection term can be inferred by conducting a regression for the final estimate as a function of the intermediate values (and their times of publication) (see Fig 2E). One could devise more intricate algorithms that take into account cross-country correlations and country-specific societal factors to correct for imperfections in surveillance.

### Surveillance data collection practices are not uniform

Even within a surveillance framework, there are systematic deviations in coverage as well as differential lags in updates. Surveillance reporting has been known to taper off or stop altogether during the post-peak part of the season. For example, as is evident from Fig 1D, the number of providers who reported to US CDC ILINet surveillance tapers off toward the end of the ILI season week (for the US, calendar week 40 corresponds to first ILI season week [4]). Specifically, the inflection point of the average curve occurs at week 33. Such effects can possibly be attributed to resource reallocation due to reduced interest in post-peak activities. This necessitates modelers to make a distinction between expected ILI/flu curves and the observed data for different parts of the season. Some efforts have been made to account for such systematic deviations by either correcting their estimates to match the surveillance reports [1] or by explicitly modeling surveillance errors [2]. More explicit data about the detailed structure of the surveillance system would benefit these corrections.

## Forecasting practices

### There is no community agreement on measure(s) of performance

Measuring forecasting skill is dependent on the actual use of the forecasts, which varies widely. Not surprisingly, there is no accepted measure of forecasting performance. In recent work [9], we have identified 7 different metrics for around 10 different quantities, each evaluating a different facet of the flu. Moreover, evaluations often involve multiple criteria, can include subjective components, and present tradeoffs in which the balance of preferences is not well articulated. A vanilla mean-squared error criterion will lead to a model with a tendency to underpredict the peaks when trained uniformly over complete seasons. However, if agencies are more interested in peak characteristics, such a measure can be modified to penalize deviations around peak more severely. Thus, understanding the requirements of health agencies is essential to generate meaningful forecasts. Additionally, quantifying forecasting uncertainty and presenting the same [9] in a meaningful manner is of prime importance to facilitate actionable strategies from health agencies.

### “Out of sample” testing is not forecasting

To the best of our knowledge, the CDC and IARPA OSI competitions are the only two competitions that truly involved forecasting into the future. Participants were required to submit time-stamped forecasts that must arrive before the event date (thus certainly before the surveillance data becomes available). In contrast, much published literature focuses on retrospective analysis on flu seasons past [10]. The benefit of hindsight affords the ability to tune models indefinitely, and out-of-sample testing is no substitute for forecasting. Dangers include not just overfitting but also vulnerability to model drift over time.

Also, it is to be noted that due to the delays and updates inherent in surveillance data, a "forecasting" project predicting 1 or 2 weeks into the future is in essence a "nowcasting" [11] system. Understanding this aspect is crucial, as it could lead to intelligent strategies for handling surrogate information [12] such as weather data and social network sensors. Since surrogate sources are typically real time, when performing nowcasting, one can ideally complement knowledge of last-known surveillance data with the current surrogates and hence find a direct estimate of the surveillance data. On the other hand, a prediction engine interested in forecasting the peak of the flu season (say 7 weeks in advance) does not have the same liberty. Some researchers have used data assimilation techniques [11, 13] to incorporate knowledge about weather data to provide ILI forecasts. Intelligent assimilation techniques can be developed to incorporate weather forecasts (which are also available) and reduce the error bounds while predicting for more than, say, 4 weeks in advance.

### More data is not always better data

There is now a wealth of syndromic surveillance and physical indicators available for forecasting the flu such as weather, news reports, web search query volumes (GFT, GST), Twitter chatter [1], and Wikipedia logs [14, 15]. Pitfalls in big data analysis without understanding the underlying system biases have been well documented (e.g., for GFT, see [16]). We evaluated diverse data sources for flu forecasting in 15 Latin American countries (Fig 2A) and have considered both data-level fusion and model-level fusion. The former concatenates all data sources and renders them in a common denominator format before modeling. Model-level fusion builds separate models with each source and combines their forecasts, leveraging selective superiorities. As Fig 2B shows, model-level fusion performs better than data-level fusion. “Ablation tests” (see Fig 2C) show that physical indicators are consistently the more important predictor than social indicators. Inclusion of some data sources can actually lead to reduced performance for specific countries (e.g., Google Search Trends for French Guiana).

### Additional resources

Data sets, associated code snippets, and abbreviations used in this article are archived at the publicly accessible github page http://prithwi.github.io/how_not2_flu.

## Conclusion

Infectious disease forecasting is a rapidly emerging field. The challenges reported here pertain to how data from surveillance systems can be modeled as well as how forecasting standards need to mature. While we have focused on flu forecasting here, these challenges translate to other diseases as well. We advocate increased efforts in fostering a community of forecasters and converging on a common understanding of shared concerns and approaches forward. We also argue for easier availability of surveillance data and surrogate sources in raw forms at all possible spatial and temporal granularities. Skilled forecasting research can significantly aid in the translation of research results into the hands of public policy professionals and decision-makers.
